# Effect of proximal *versus* distal 50% enterectomy on nutritional parameters in rats preconditioned with a high-fat diet or regular chow

**DOI:** 10.1038/srep17331

**Published:** 2015-11-27

**Authors:** Ujwal R. Yanala, Roger D. Reidelberger, Jon S. Thompson, Valerie K. Shostrom, Mark A. Carlson

**Affiliations:** 1 Department of Surgery, University of Nebraska Medical Center, Omaha, NE 68198; 2 Department of Surgery, VA-Nebraska Western Iowa Health Care System, Omaha, NE 68105; 3 Department of Biomedical Sciences, Creighton University, Omaha, NE 68178; 4 Department of Biostatistics, University of Nebraska Medical Center, Omaha, NE 68198; 5 Department of Genetics, Cell Biology, and Anatomy, University of Nebraska Medical Center, Omaha, NE 68198.

## Abstract

Obesity may protect against the nutritional consequences of short bowel syndrome. We hypothesized that rats preconditioned with an obesogenic diet would have better outcomes after surgical induction of short bowel syndrome compared to rats on regular chow. Rats were fed a high-fat diet or regular rat chow for six months, and then underwent 50% proximal, 50% distal, or sham enterectomy. Food intake, weight, and body composition were monitored before and for 4 weeks after surgery. The high-fat diet consistently produced obesity (>25% body fat). All procedures induced weight loss, but there was no discernable difference between resection *vs*. sham resection. Rats on the high-fat diet had a greater post-resection loss of body fat compared to rats on chow (36 *vs*. 26 g, respectively). There was a nonsignificant trend of less lean mass loss in the former compared to the latter rats (16 *vs*. 33 g, respectively). Enterectomy moderated serum ghrelin, GIP, PPY, insulin, and leptin. Intestinal adaptation was not different between obese *vs.* non-obese rats. Rats preconditioned with the high-fat diet may have had better retention of lean body mass after a surgical procedure compared to rats on chow. The effect of 50% enterectomy was less than expected.

Clinical data have suggested that obese patients (BMI > 35) tolerate Short Bowel Syndrome (SBS) better than non-obese patients^1^. Specifically, obese patients tended to retain their initial weight after extensive small intestinal resection, as compared to non-obese patients. The physiologic basis for this finding was not clear. These findings were corroborated in a review of bariatric patients, in which weight loss induced by gastric bypass appeared to eliminate the benefits of obesity in patients who subsequently developed SBS^2^. Documentation and understanding of a protective effect of pre-existing obesity on outcomes after massive intestinal loss could be helpful in the management of patients with SBS. Although the precise incidence is difficult to determine, it has been estimated that about 25 patients per million inhabitants of Western countries have SBS with intestinal failure (i.e., with dependence on parenteral nutrition)^3^.

Murine studies of extensive intestinal resection have suggested that the amount of fat in a postoperative diet and/or a post-resectional hyperphagic mechanism might account for the retention of body weight, with the increased caloric intake compensating for the loss of digestive and absorptive area of the intestine^4^. Our intention with the present study was to determine whether rats with pre-existing obesity (induced with a high-fat diet) would have improved outcomes after massive intestinal resection compared to non-obese rats. We hypothesized that the presence of obesity in the rat would be protective of body weight and other nutritional endpoints after massive intestinal resection. If the presence of obesity did influence post-resectional outcomes, then a secondary intention of this study was to gain a preliminary insight into the mechanism of the obesity effect.

## Results

**Figure 1:**
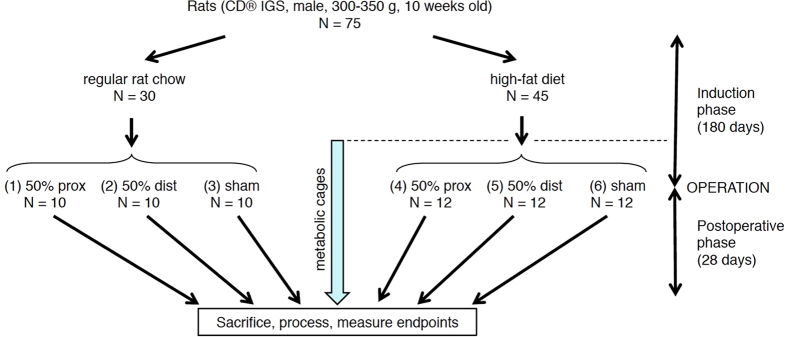
Experimental flowchart. 50% prox = resection of proximal half of small intestine; 50% dist = resection of distal half; sham = transection and anastomosis of mid-small intestine, without resection.

**Figure 2:**
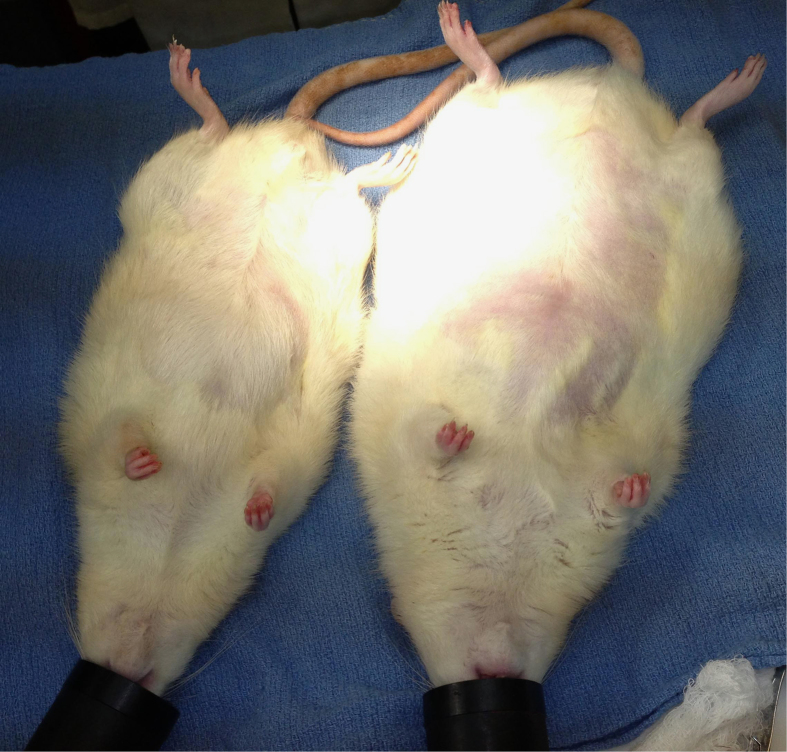
Lean *vs*. obese rat subjects. Images taken just prior to necropsy, 30 days after resection. Left subject = regular chow; right = high-fat diet.

An overview of the experimental design for this study is shown in Fig. 1. The rats as ordered from the vendor weighed 300–350 g (9 weeks old) on arrival; they underwent a one week acclimatization period prior to initiation of the study diets. After 180 days of a regular rat chow *vs*. a high-fat diet, the mean mass of the rats were 705 ± 79 *vs*. 921 ± 125 g, respectively (Supplementary Table S2); p < 0.0001, unpaired t-test. Nonparametric testing suggested that diet type had a significant effect on preoperative weight (Table 1, row 1), consistent with the above t-test of rats grouped by diet type. The relative size of a typical rat on either diet is shown in Fig. 2.

QMR analysis at 165 days of assigned diet (induction phase) demonstrated mean fat mass of 124 ± 41 *vs*. 289 ± 61 g, regular rat chow *vs*. high-fat diet respectively, with %body fat of 17.6 ± 4.3 *vs*. 32.1 ± 4.7%; p < 0.0001 for both comparisons (unpaired t-test; Supplementary Table S3). These QMR data subsequently were used to stratify the rats into procedure groups (see Methods). Nonparametric testing suggested that diet type had a significant effect on both preoperative fat mass and %body fat (Table 1, rows 4 and 5), consistent with the above t-tests of rats grouped by diet type.

QMR-determined lean mass at 165 days trended higher in the high-fat compared to the regular chow rats (502.4 ± 47.3 *vs*. 480.0 ± 41.2 g, respectively; p = 0.0594), but the %lean mass was predictably higher in the latter group (56.4 ± 4.5 *vs*. 69.4 ± 3.9%, respectively; p < 0.0001, unpaired t-tests); see Supplementary Table S3. Nonparametric testing indicated that diet type did not have a significant effect on lean mass (Table 1, row 8), consistent with the above t-test.

During the 180 day induction phase, two rats expired, one in each dietary group (Supplementary Table S2); the cause of death was not determined in these two subjects. The intestinal resection procedures were begun 180 days after initiation of the study diets, and were completed over four consecutive days. Post-resection mortality secondary to anastomotic leak/intraabdominal sepsis occurred in two *vs*. eight rats (7 *vs*. 18%) in the regular chow *vs*. high-fat diet groups, respectively (Supplementary Table S2); p = 0.153, Fisher Exact Test. One rat in the regular chow group did not undergo the final set of measurements because of an administrative error. With these eliminations, 27 and 36 rats were left for necropsy and final analysis in the regular chow *vs*. high-fat diet groups, respectively. None of these rats in the final analysis appeared to have an anastomotic stricture at necropsy, as evident by a lack of obvious pre-anastomotic intestinal dilatation.

**Figure 3:**
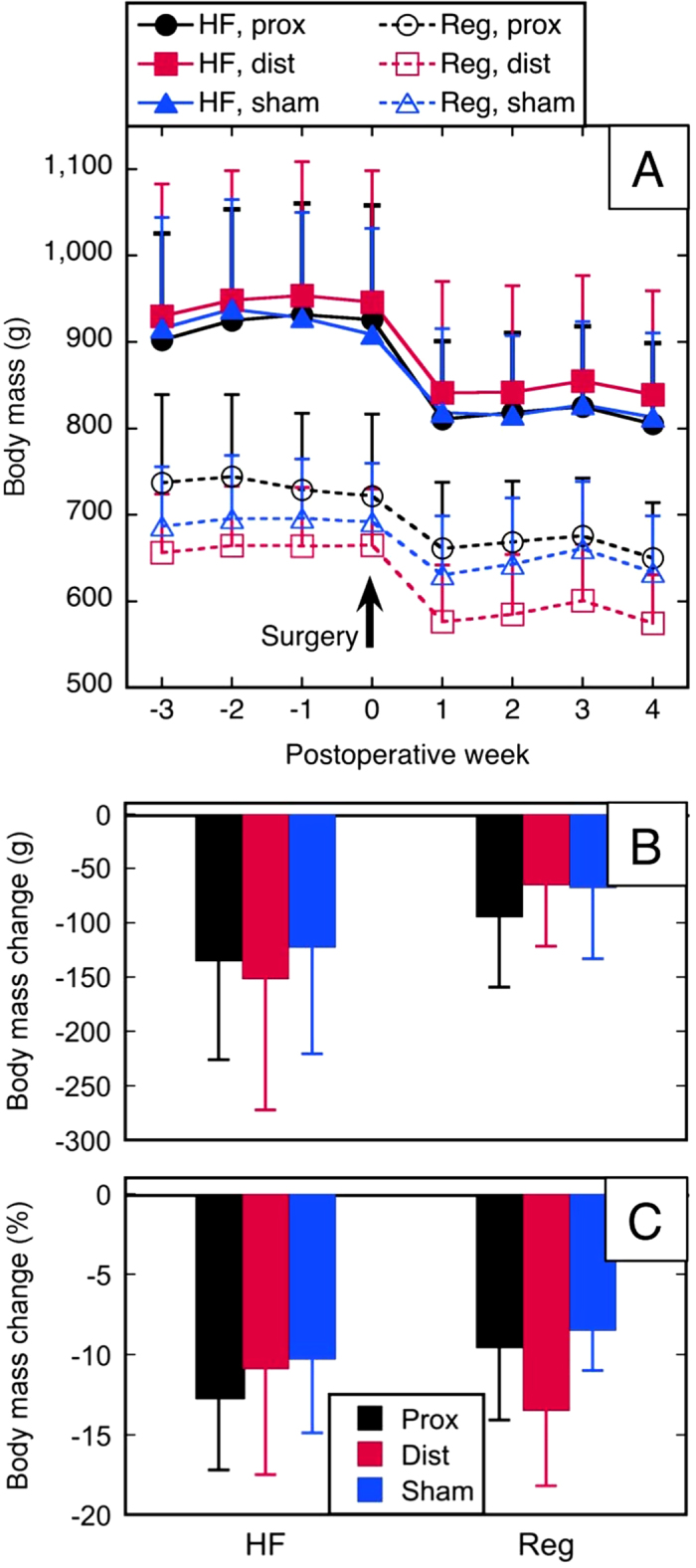
Change in body mass. (**A**) Body mass *vs*. time; (**B**) Change in body mass (pre-resection mass minus pre-necropsy mass); (**C**) percent change in body mass. HF = high-fat diet; Reg = regular chow; prox, dist, sham = 50% proximal, 50% distal, or sham resection, respectively.

**Figure 4:**
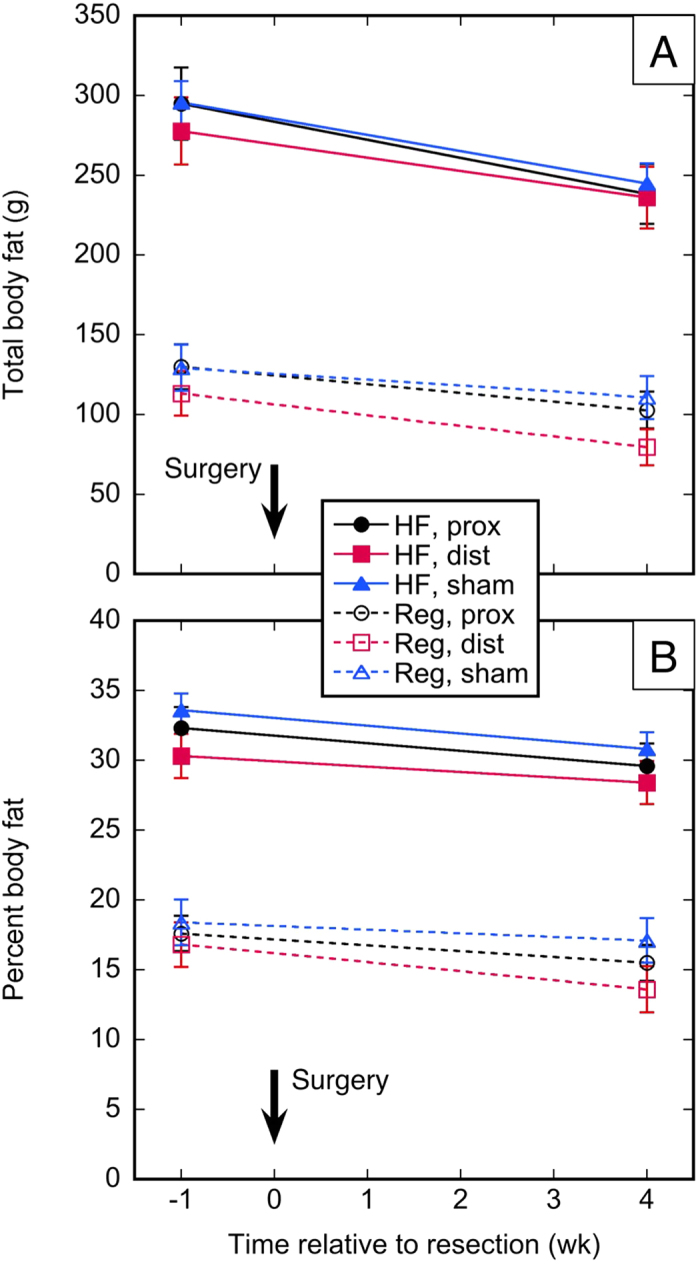
Change in body fat. (**A**) Body fat *vs*. time; (**B**) Percent body fat *vs*. time. HF = high-fat diet; Reg = regular chow; prox, dist, sham = 50% proximal, 50% distal, or sham resection, respectively.

Necropsy performed in the subjects that survived Summary statistics of body weight and composition before and after resection are shown in Supplementary Table S3 and Figs 3, 4. All three intestinal procedures (50% proximal enterectomy, 50% distal enterectomy, and sham/simple transection with anastomosis) in both diet types produced weight loss in the first week after the procedure (Fig. 3A); body weight then plateaued in all six groups for the duration of the one month post-procedure observation period. The subjects did not regain their lost weight. Nonparametric testing demonstrated, however, that weight loss was not different among the six treatment groups (Table 1, rows 2 and 3).

Body fat of the subjects on the high-fat diet decreased from 289 ± 61 g (pre-resection) to 253 ± 66 g (necropsy); respective values for subjects on regular chow were 124 ± 41 g to 98 ± 37 g (p < 0.0001 for both diet types, paired t-test; see Supplementary Table S3 and Fig. 4). The delta between the above pre-resection and necropsy body fat means was 36 *vs*. 26 g, respectively; i.e., the rats on the high-fat diet appeared to lose more fat after resection. The fraction of subjects exceeding 30% body fat in the high-fat diet group were 24/36 (pre-resection) and 23/36 (necropsy); in the regular chow group, no subject exceeded 30% body fat (Supplementary Table S3). Nonparametric testing of the change in body fat mass (but not %body fat) from pre-resection to necropsy among the six groups indicated that changes in fat mass were not equivalent (Table 1, rows 6 and 7). Subsequent pairwise testing did not demonstrate an effect of resection type on body fat mass, but did demonstrate that rats on a high-fat diet lost more body fat than rats on regular chow (p < 0.0001; Supplementary Table S3).

Lean mass of the subjects on the high-fat diet decreased from 502 ± 47 g (pre-resection) to 486 ± 47 g (necropsy); respective values for subjects on regular chow were 480 ± 41 g to 447 ± 37 g (p < 0.0001 for both diet types, paired t-test; see Supplementary Table S3). Although the rats on the high-fat diet appeared to lose less lean mass than the rats on regular chow (a respective delta in the above means of 17 *vs*. 33 g), nonparametric testing of the change in lean mass (and also the change in %lean mass) from pre-resection to necropsy among the six groups indicated that no statistical differences were present (Table 1, rows 9 and 10).

QMR data on the contribution of either water mass or active tissue mass to the total lean mass are given in Supplementary Table S4. The relative contributions of the change in water mass or active tissue mass to the change in total lean mass from the preoperative time point to necropsy in all six groups was 90 and 10%, respectively (Supplementary Table S4, worksheet 5). There was no significant difference in the contribution of either water or active tissue to total lean mass prior to the resection among all six groups (Supplementary Table S4, worksheets 3 and 4). At necropsy, subjects on regular chow and undergoing a distal resection had lower contributions of both water and active tissue to total lean mass compared to subjects undergoing any resection while on a high-fat diet, suggesting that the high-fat diet was protective of lean mass at this single time point. The change over time in contribution of either water or active tissue to total lean mass from the preoperative time point to necropsy was not different among all six groups.

**Figure 5:**
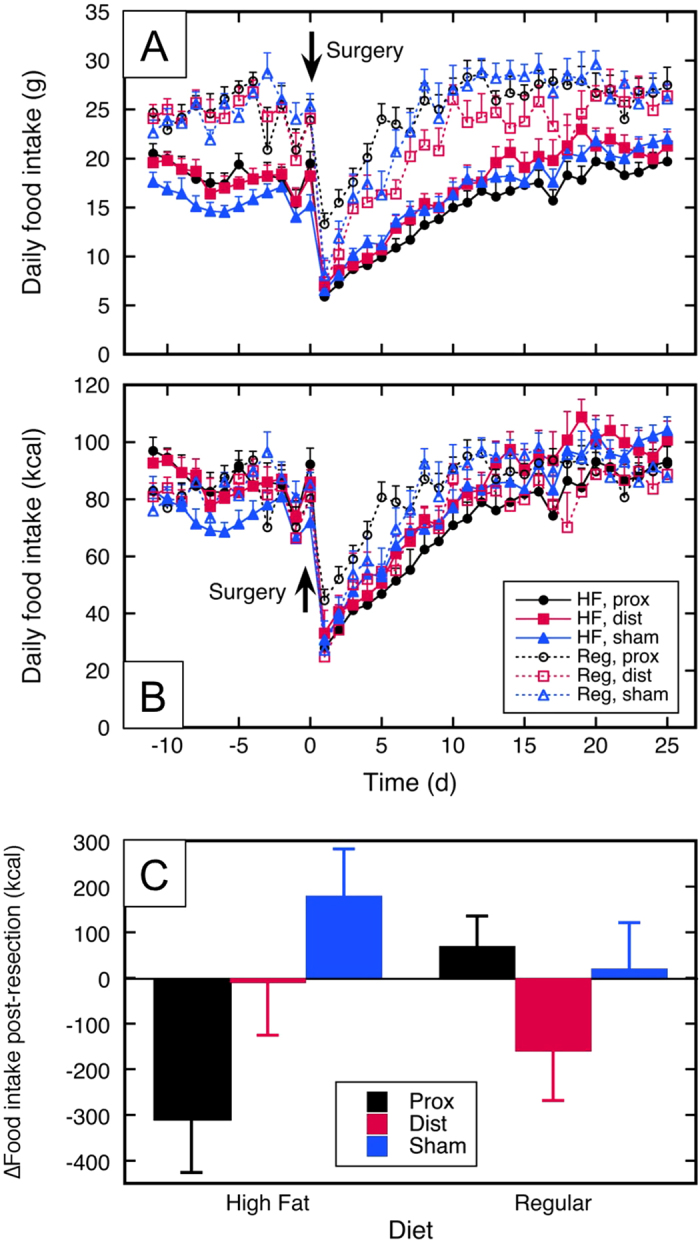
Food Intake. (**A**) Food intake (grams/day *vs.* time) *vs*. time; (**B**) Food intake (kcal/day *vs.* time) *vs*. time; (**C**) Cumulative change in food intake post-resection (see text). HF = high-fat diet; Reg = regular chow; prox, dist, sham = 50% proximal, 50% distal, or sham resection, respectively.

Daily food intake on day 1 post-resection decreased to 20–40% of the pre-resection amount in all groups (Fig. 5A,B). The effect of surgical procedure and diet on the post-resection change in cumulative food intake is shown in Fig. 5C and Table 1 (row 11). Nonparametric testing of the post-resection change in cumulative food intake among the six groups indicated that the values were not equivalent (Table 1). Pairwise testing indicated that in subjects on a high-fat diet, proximal resection resulted in a lower food intake than sham resection; other comparisons were nonsignificant.

A summary of villus height and muscular externa measurements at time of sacrifice is shown in Table 2; the complete dataset is contained in Supplementary Table S5, and typical images for the S4 segment are shown in Supplementary Figure S4. Comparing villus heights of different intestinal segments within the same treatment group, villus height was lower in the distal intestinal segment (S4) compared to the proximal segments among subjects undergoing a sham resection (Table 2). This lower S4 villus height was not present in subjects undergoing enterectomy, except in subject on a high-fat diet with distal enterectomy. Comparing villus height from the same segment among all treatment groups, the mid-intestinal segment (S2) villus height was modestly but significantly greater in the high-fat sham resection group *vs*. the regular chow sham resection group (Table 2). In addition, the S4 segment villus height was 30–40% greater in the enterectomy groups compared to the sham groups.

The thickness of the muscularis externa of the S1-S4 segments was fairly uniform within a given treatment group, except within the high-fat sham resection subjects, in which the thickness values from different segments were significantly different (Table 2). Comparing the thickness of the muscularis externa from the same segment among all treatment groups, thickness in the S1 segment appeared to increase in the regular chow rats after either proximal or distal enterectomy compared to sham resection; this effect was not obvious in the rats on the high-fat diet (Table 2). Thickness in the S4 segment also increased after proximal or distal resection compared to sham resection, perhaps more so in the rats on regular chow.

A summary of gastrointestinal peptide plasma levels at the time of sacrifice, with statistical testing results, is shown in Supplementary Table S6. The GLP-1 level was too low to determine in all subjects. No significant differences were noted for amylin. Resection type appeared to affect the ghrelin level per ANOVA testing, but unpaired t-testing with sham did not reveal a significant effect of proximal or distal resection. Both diet and resection type had an effect on the GIP level; subjects on a high-fat diet had higher levels (30–50% increase within a given resection type), and both proximal and distal resection resulted in GIP decrease (~50%) compared to sham. The plasma level of insulin and leptin in the rats on the high-fat diet were 1.3-fold and 2–3 fold the values in the rats on regular chow, respectively. Proximal or distal resection approximately doubled the PYY level over sham resection, regardless of diet type.

## Discussion

In the field of cardiovascular research, an “obesity paradox” has been observed in which obese patients suffering from coronary artery disease (or other cardiovascular illness) actually may have better outcomes than non-obese patients^5^. The paradox, of course, is because obesity also is a risk factor for coronary artery disease. A protective effect of obesity has been postulated for all-cause mortality in the elderly^6^, mortality after non-bariatric general surgical and oncologic operations^7^,^8^, cancer survival^9^, and mortality in critically ill patients^10^. The mechanism of the obesity paradox is under investigation^11^,^12^, and the definition of this paradox still is disputed, because of the long-established association of obesity with cancer, cardiovascular disease, diabetes, and overall mortality^13^,^14^. It is possible that obesity is a marker for a better nutritional state in patients with chronic disease, thereby leading to better outcomes compared to non-obese/underweight patients with the same chronic disease^12^.

In this study we attempted to determine whether evidence existed for the obesity paradox in an experimental model of short bowel syndrome. That is, we investigated in rats whether pre-existing obesity (induced with a high-fat diet) would have an effect on nutritional endpoints after massive small intestinal resection. In overview, we found that while the “obese” rats lost more body weight and fat after a surgical procedure than the non-obese rats, there was a suggestion (nonsignificant) that lean mass was better preserved in the obese rats. This latter finding is consistent with an “obesity paradox,” i.e., the obese rats may have had a superior outcome in an important nutritional endpoint (lean body mass) compared to the non-obese rats. Regarding previous definitions of obesity in experimental rat studies, a percent body fat of >20 or 25% have been used^15^,^16^,^17^; by this criteria, the rats on the high-fat diet in the present study were obese at the time of resection, having mean percent body fat in excess of 30% (Table 1).

One predicted outcome not observed in this study was a consistent differential effect of the three surgical procedures. For instance, there did not appear to be an effect of sham resection *vs.* 50% proximal enterectomy *vs.* 50% distal enterectomy on body weight, percent body fat, or lean mass. Possible reasons for this observation of no effect include (1) too short of a postoperative observation period; or (2) an inadequate length of intestinal resection. That is, with a longer postoperative observation period and/or a greater length of intestinal resection, significant differences between sham resection and enterectomy groups may become obvious.

On a similar note, a 30-day post-resection observation period may have been inadequate to observe differential effects of intestinal adaptation that can occur after massive intestinal resection^18^. There was some evidence of adaptation in terms of both villus height and thickness of muscularis externa that occurred in the enterectomy groups relative to the sham groups, but these differences were relatively small, and an effect of obesity was not obvious. Of note, histologic measurements represent a single time point (sacrifice), so determination of adaptation was done by comparing sham with resected groups. Additional histologic measurements at time zero (i.e., the resection day), which would have allowed each subject to serve as their own control, were not obtained.

Another variable that might have contributed to relative differences observed in this study was the postoperative diet. All subjects were maintained on the same diet that they had been assigned to preoperatively, i.e., obese rats were kept on the high-fat diet, and non-obese rats were kept on regular chow. Previous work on intestinal adaptation after resection demonstrated that a high-fat postoperative diet enhanced villus length and smooth muscle contraction with respect to a low-fat diet^4^,^19^,^20^. So there may have been the possibility that the different postoperative diets influenced intestinal adaptation independent of preoperative body composition. In order to control for the possible influence of postoperative diet on intestinal adaptation, it may have been better in our study to have administered the same diet in all subjects after the resections.

Serum hormone levels at time of sacrifice were collected in this study in order to gain insight into possible mechanisms for any observed effects. The effects on ghrelin were minor, but the trend was for lower levels after proximal resection and higher levels after distal resection, with no effect of obesity. Interestingly, the rats on the high-fat diet ate less after a proximal resection relative to the rats on regular chow, while the former subjects appeared to eat more relative to the latter subjects after distal resection; these observations may be related to the changes in ghrelin levels. Previous work in rats demonstrated that ghrelin is secreted from the stomach to the colon^21^, and that ghrelin increases acutely (within one day) after massive small intestinal resection^22^; this study may not be directly comparable with our study, since we used a later time point (30 days) to assay ghrelin.

The increase of GIP observed in subjects on a high fat diet in our study was consistent with previous rodent studies^23^, and with GIP’s known role in the induction of insulin in obese subjects. The explanation for decreased GIP after a resectional procedure (proximal or distal) compared to sham likely is due to removal of a source of GIP secretion. Interestingly, PYY was elevated after either proximal or distal resection compared to sham; PYY has a known inhibitory effect on appetite^24^, but also serves as a brake on motility (thus perhaps increasing digestive efficiency)^25^. The large intestine also is a significant source of PYY^26^. Thus, a likely increase in delivery of chyme to the large intestine after small bowel resection may increase PYY release from the large intestine. Such a mechanism has been hypothesized for the large increase in PYY levels observed after gastric bypass^27^,^28^. So the role PYY is playing in modulation of body composition 30 days after 50% enterectomy in the rat is not clear. The increases of both insulin and leptin observed in our subjects on a high fat diet are consistent with the known biology of these peptides^29^,^30^.

This study suggested (but did not demonstrate) that pre-existing obesity was protective of the nutritional state in surgically-induced SBS. Regarding future experimentation, it may be worthwhile to repeat this type of study using an enterectomy of a greater length (that is, more than 50%) of the small bowel and a longer (more than one month) postoperative observation period. In addition, a possible confounding effect may be controlled if all rats are placed on the same diet after surgical intervention. In the present study, each rat was maintained on its preoperative diet after surgery, which added an additional variable during the postoperative observation period. A new study with the above modifications may better illuminate the hypothesized protective effects of obesity on nutritional parameters in experimental SBS.

## Materials and Methods

### Animal Welfare

This animal research study was carried out in accordance with recommendations in the *Guide for the Care and Use of Laboratory Animals* (8^th^ ed.) from the National Research Council and the National Institutes of Health^31^. The animal protocol was approved by the Institutional Animal Care and Use Committee of the VA Nebraska-Western Iowa Health Care System (protocol number 00834), and by the Institutional Animal Care and Use Committee of the University of Nebraska Medical Center (protocol number 12-097-11-ET). All procedures were performed in animal facilities approved by the Association for Assessment and Accreditation of Laboratory Animal Care International (AAALAC; www.aaalac.org) and by the Office of Laboratory Animal Welfare of the Public Health Service (http://grants.nih.gov/grants/olaw/olaw.htm).

All surgical procedures were performed under isoflurane anesthesia, and all efforts were made to minimize suffering. Euthanasia was performed in accordance with the AVMA Guidelines for the Euthanasia of Animals^32^. In addition, this study was conceived, designed, documented, analyzed, and reported per the ARRIVE (Animal Research: Reporting of *In Vivo* Experiments) Guidelines^33^; the ARRIVE Guidelines Checklist is shown in Supplementary Table S1.

### Animal Subjects and Determination of Subject Numbers

CD® IGS rats (nomenclature Crl:CD(SD); males; age = 10 weeks at beginning of induction phase; see Fig. 1) were purchased from Charles River Laboratories International, Inc. The minimum number of rats (n = 8) utilized for each experimental group in Fig. 1 was determined with a statistical power analysis^34^ using Δ/σ (Cohen’s *d*, in which Δ is the desired difference in means as set by the observer, and σ is the estimated standard deviation) = 1.75, false positive rate (α) = 0.05, false negative rate (β) = 0.2, and power (1–β) = 0.8. The predetermined endpoints of interest in this study were body mass, composition, and intestinal dimensions. The actual number of subjects per group at the beginning of the induction phase in Fig. 1 was increased to 10 and 15 for experimental groups 1–3 and 4–6, respectively. The justification for these increased subject numbers was in case of subject loss secondary to illness or perioperative mortality or, for groups 4–6, in case some subjects did not have adequate weight gain on the high-fat diet (see Results).

### Experimental Design Overview

Refer to Fig. 1 for an experimental flow diagram. Rats (N = 75 at start) were fed either regular chow (N = 30) or a high-fat diet (N = 45) for 180 days, and then underwent enterectomy (sham resection, 50% proximal resection, or 50% distal resection). Subjects were maintained on the same diet postoperatively that they had been on preoperatively; sacrifice with final endpoint measurements were performed 30 days after enterectomy.

### Induction Phase

Per Fig. 1, 30 rats were randomly assigned to be fed *ad libitum* with regular rat chow (LabDiet 5001 Rodent Diet; PMI Nutrition® Inc.) and water, and 45 rats were assigned to be fed *ad libitum* with a high-fat diet (OpenSource Diets® product no. D12451; Research Diets™, Inc.) and water; for a summary of dietary formulations, see Supplementary Figure S1. Rats were housed in pairs during all but the last ten days of the induction phase, and were weighed once per week until sacrifice. Total lean and fat masses were determined noninvasively with quantitative magnetic resonance (QMR) once per month throughout the induction phase (EchoMRI™ 700 Whole Body Composition Analyzer; Echo Medical Systems).

Ten days prior to the surgical procedure, each rat was housed individually in a metabolism cage (catalog no. 5236, Lab Products Inc., Maywood, NJ) modified to include a rectangular stainless steel side compartment (25.4 cm long × 17.8 cm wide × 17.8 cm high) with a round hole (3 cm diameter) in the base. Below the hole was a food cup fixed to a digital scale. Output from each scale was monitored by computer at ~20 s intervals, and changes in food cup weight were recorded and processed to determine daily food intake until sacrifice^35^. A 12 h light/dark cycle (lights on at 1100 h) was maintained for the entire study.

At the end of this adaptation period, lean and fat masses were again determined using QMR. The 30 rats fed *ad libitum* on regular rat chow were divided into three groups of 10 rats (groups 1–3) matched for percentage body fat, and the 45 rats fed *ad libitum* on high-fat diet were divided into three groups of 15 rats each (groups 4–6) matched for percentage body fat (see Fig. 1). Group assignment was done on the basis of body fat (as opposed to a randomized process) to ensure that the distribution of body fat values among groups in each cohort (regular chow or high-fat diet) was comparable.

### Surgical Procedure

Rats were fasted for 24 h preoperatively, but with free access to water. Inhalational isoflurane was administered for anesthesia, and cefovecin (20 mg; a long acting cephalosporin^36^) was injected subcutaneously. The subject was placed supine, and the abdomen was shaved, washed with antiseptic solution (Povidone Iodine Topical Solution, 10% USP, Allegiance, Cardinal Health), and the surgical site was isolated with sterile adhesive drapes. A ventral vertical midline incision was made, extending from the xiphoid process caudally to just superior to the urethral meatus. The length of the small intestine from the pylorus to the cecum was measured using a cotton umbilical tape. Intestinal surfaces were kept moist during the procedure with saline-soaked gauze.

For the 50% proximal enterectomy, the small intestine was resected several cm distal to the pylorus to the midpoint of the small intestine, as determined with the umbilical tape measurement. Ligatures of 4-0 silk were used to control mesenteric vessels. An end-to-end small intestinal anastomosis was performed using a single layer of continuous 6-0 silk (Ethicon Perma-Hand® Silk, catalog no. K889H). For the 50% distal enterectomy, the small intestine was resected from the midpoint of the small intestine to several cm proximal to the cecum, preserving the ileocecal valve, followed by the anastomosis. For the sham resection, the small intestine was transected at its midpoint without any resection, followed immediately by the anastomosis.

The abdominal musculofascial layers then were closed in one layer with continuous 3-0 polyester suture (Ethibond Excel®, Ethicon, Inc., Johnson & Johnson; catalog no. X558H), followed by staples (Autoclip® 9 mm stainless steel clips; MikRon Precision, Inc., catalog no. 427631) to close the skin. Antibiotic ointment (Fura-Zone, 0.2% nitrofurazone; Squire Laboratories, Inc.) was applied once along the skin staple line. Buprenorphine (0.1 mg) then was injected into a thigh muscle, and 10 mL of saline was injected intraperitoneally for hydration.

### Postoperative Phase

After the surgical procedure, rats were transferred to their metabolic cages and were supplied food and water *ad libitum*, each continued on the same diet from the preoperative period. After 28 days of postoperative data collection (feeding & weights), each rat was weighed and then anesthetized with isoflurane. The ventral midline incision was re-opened, the diaphragms were opened bilaterally, and a blood specimen for plasma peptide analysis was collected by cardiac puncture with a 22 g needle. The subject then was exsanguinated with cardiotomy, and both diaphragms were incised. The small intestine from the stomach to the cecum was resected *en bloc*, grossly inspected, measured for length, and photographed. Segments (1 cm long) of intestine were harvested as shown in Supplementary Figure S2, and were fixed in buffered 10% formalin for histology. The carcass then was subjected to QMR for final body composition determination.

### Endpoints

Body weight, mass of daily food intake, and the body composition data were captured as described above. Plasma peptide levels (ghrelin (active), glucose-dependent insulinotropic peptide (GIP total), glucagon-like peptide-1 (GLP-1 active), insulin, leptin, peptide YY (PYY), and amylin (active)) were measured from the terminal blood specimen using a multiplex assay (Rat Metabolic Magnetic Bead Panel Kit, catalog no. RMHMAG-84K, Millipore Corp., St. Charles, MO). After an overnight fast, blood samples (3–4 ml) were collected by cardiac puncture into EDTA tubes containing 40 μL of a mixture of 0.9 mL DPP IV inhibitor (Linco™, catalog no. DPP4), 0.1 mL Protease Inhibitor Cocktail (Sigma, catalog no. P2714), and 0.1 g AEBSF (Roche Pefabloc®, catalog no. 11 429 868 001). Tubes were stored on ice for ~30 min, then centrifuged at 3000 *g*, and plasma was separated and stored at −70 °C.

Individual plasma samples were extracted using the OASIS HLB LP 96-well extraction plate (Waters Corp., Milford, MA, USA)^37^ before the peptide multiplex assay was performed. Digital micrographs of intestinal sections (paraffin-embedded, H&E stained) were captured using an EVOS® XL Core microscope (Advanced Microscopy Group, Life Technologies). Intestinal dimensions including thickness of muscularis externa and villus height were measured from the micrographs using ImageJ software (http://imagej.nih.gov/ij/); see Supplementary Figure S3 for definitions used in these measurements.

### Statistical Analysis

PC SAS version 9.4 (http://www.sas.com) was used for all analyses. For each variable of interest, a nonparametric Kruskal-Wallis test was used to compare the six combinations of diet and surgical procedure category. The six combinations were: regular/sham, regular/proximal, regular/distal, high-fat/sham, high-fat/proximal, and high-fat/distal. If this test was statistically significant, then pairwise comparisons were made.

## Additional Information

**How to cite this article**: Yanala, U. R. *et al.* Effect of proximal *versus* distal 50% enterectomy on nutritional parameters in rats preconditioned with a high-fat diet or regular chow. *Sci. Rep.* **5**, 17331; doi: 10.1038/srep17331 (2015).

## Supplementary Material

Supplementary Information

## Figures and Tables

**Table 1:** Body weight and body composition.

Row	Variable	Reg, Sham(n = 9)	Reg, Prox (n = 9)	Reg, Dist (n = 8)	HF, Sham (n = 12)	HF, Prox (n = 11)	HF, Dist (n = 13)	Overall *p-value	*Pairwise p (Reg)		*Pairwise p (HF)	
									Sham/Prox	Sham/Dist	Sham/Prox	Sham/Dist
1	iBW (g)	678 (648, 722)	731 (663, 800)	648 (614, 705)	878 (844, 931)	946 (838, 1015)	955 (857, 1064)	**<0.0001**	0.54	0.41	0.48	0.34
2	∆BW (g)	−58 (−72, −51)	−85 (−91, −59)	−73 (−128, −66)	−91 (−117, −63)	−108 (−162, −79)	−94 (−118, −67)	0.0644				
3	∆BW (%)	−8.3 (−11, −7.0)	−11 (−12, −9.9)	−12 (−18, −10)	−9.7 (−13, −7.1)	−10 (−17, −9.5)	−8.8 (−14, −7.3)	0.2264				
4	iFat (g)	132 (104, 157)	134 (93, 140)	109 (87, 123)	294 (252, 337)	309 (243, 346)	289 (216, 346)	**<0.0001**	0.99	0.36	0.75	0.69
5	i%BF	19 (16, 22)	17 (15, 19)	17 (14, 19)	34 (29, 37)	34 (29, 36)	31 (25, 36)	**<0.0001**	0.79	0.53	0.60	0.15
6	∆Fat (g)	−17 (−24, −10)	−28 (−33, −22)	−24 (−58, −16)	−48 (−81, −33)	−46 (−91, −39)	−30 (−71, −21)	**0.0086**	0.25	0.14	0.60	0.47
7	∆Fat (%)†	−13 (−23, −8.6)	−20 (−28, −14)	−27 (−39, −16)	−13 (−26, −12)	−19 (−27, −15)	−14 (−26, −8)	0.2140				
8	iLean (g)	466 (454, 501)	513 (484, 535)	467 (428, 498)	488 (472, 518)	499 (483, 515)	537 (450, 575)	0.1347				
9	∆Lean (g)	−25 (−34, −16)	−42 (−47, −23)	−45 (−53, −21)	−23 (−28, −13)	−22 (−37, −7.2)	−20 (−32, −5.1)	0.1523				
10	∆Lean (%)†	−5.3 (−6.5, −3.6)	−7.7 (−9.2, −4.6)	−9.7 (−11, −4.8)	−4.7 (−6.2, −2.7)	−4.7 (−6.7, −1.5)	−3.7 (−6.5, −1.2)	0.0689				
11	∆Intake (kcal)	−71 (−168, 95)	−167 (−172, 80)	177 (−90, 401)	−167 (−396, 75)	239 (−9, 719)	−6 (−126, 71)	**0.0406**	0.86	0.27	**0.01**	0.24

Initial (i) values represent measurements taken immediately prior to operation; delta (∆) values represent the difference between the necropsy and initial measurements.

^†^Percentages represent the delta value divided by the initial value. BW = body weight; Fat = fat mass; %BF = percent body fat; Lean = lean mass; ∆Intake = cumulative change in caloric intake during postoperative observation period relative to the preoperative period; Reg = rats fed regular rat chow; HF = rats fed high-fat diet; Sham = sham resection; Prox = 50% proximal enterectomy; Dist = 50% distal enterectomy. Each value is given as the median with the 25^th^ and 75^th^ percentiles in parentheses.

^*^Kruskal-Wallis test (significant results in bold font/high-lighted in yellow); pair-wise comparisons performed if overall p-value was <0.05.

**Table 2:** Summary of histologic measurements of small intestine.

Villus Height							*p-value, among groups
Segment	Group 1 (Reg Sham)	Group 2 (Reg Prox)	Group 3 (Reg Dist)	Group 4 (HF Sham)	Group 5 (HF Prox)	Group 6 (HF Dist)	
S1	842 ± 85	998 ± 137	880 ± 120	829 ± 102	920 ± 131	920 ± 135	0.0656
S2	758 ± 149	NA	NA	878 ± 73	NA	NA	**0.0390**
S3	768 ± 110	NA	NA	879 ± 152	NA	NA	0.0986
S4	570 ± 94	979 ± 150	782 ± 48	596 ± 117	1,005 ± 161	814 ± 127	**<0.0001**
*p-value, within group	**0.0012**	0.7003	0.0641	**0.0004**	0.2207	**0.0387**	
Muscularis Externa							
Segment	Group 1 (Reg Sham)	Group 2 (Reg Prox)	Group 3 (Reg Dist)	Group 4 (HF Sham)	Group 5 (HF Prox)	Group 6 (HF Dist)	*p-value, among groups
S1	220 ± 28	307 ± 51	340 ± 57	201 ± 31	263 ± 59	235 ± 46	**<0.0001**
S2	288 ± 72	NA	NA	235 ± 46	NA	NA	0.1052
S3	240 ± 39	NA	NA	239 ± 41	NA	NA	0.9342
S4	222 ± 36	322 ± 50	310 ± 41	177 ± 25	241 ± 37	264 ± 36	**<0.0001**
*p-value, within group	0.1094	0.6304	0.2076	**0.0004**	0.4142	0.1147	

See Supplementary Table S5 for complete listing of data and statistical testing. Reg = regular rat chow; HF = high-fat diet; Sham = transection & anastomosis without resection; Prox = 50% proximal enterectomy; Dist = 50% distal enterectomy; S1–S4 segment definition given in Methods; NA = not available.

^*^Kruskal Wallis nonparametric testing was used to generate p-values. All dimension are in μm, and given as mean ± standard error. Significant tests are in bold font/high-lighted in yellow.
